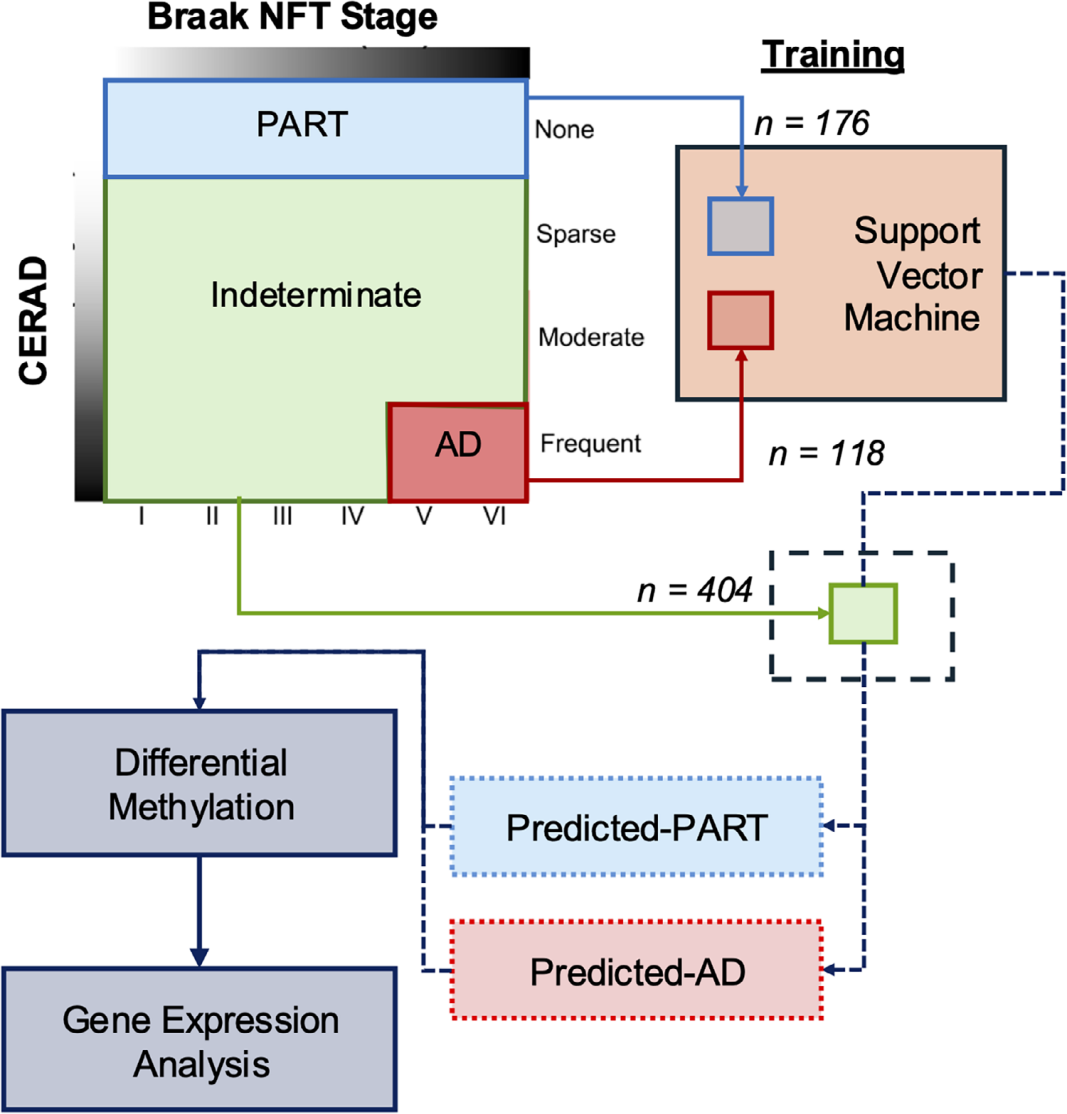# Classifying AD and PART: An Epigenetic Signature of Cognitive Resilience

**DOI:** 10.1002/alz70855_107010

**Published:** 2025-12-24

**Authors:** Anil R. Wadhwani, David C. Goldberg, Philip L. De Jager, David A. A. Bennett, David A. Wolk, Eddie B Lee, Kurt Farrell, John F. Crary, Wanding Zhou, Corey T. McMillan

**Affiliations:** ^1^ Department of Neurology, University of Pennsylvania Perelman School of Medicine, Philadelphia, PA, USA; ^2^ Center for Computational Genomic Medicine, Children's Hospital of Philadelphia, Philadelphia, PA, USA; ^3^ Department of Neurology and the Taub Institute for the Study of Alzheimer's Disease and the Aging Brain, Columbia University Irving Medical Center, New York, NY, USA; ^4^ Rush Alzheimer's Disease Center, Rush University Medical Center, Chicago, IL, USA; ^5^ Department of Neurological Sciences, Rush University Medical Center, Chicago, IL, USA; ^6^ Institute on Aging, University of Pennsylvania, Philadelphia, PA, USA; ^7^ Department of Neurology, Perelman School of Medicine, University of Pennsylvania, Philadelphia, PA, USA; ^8^ Penn Memory Center, University of Pennsylvania, Philadelphia, PA, USA; ^9^ Department of Pathology & Laboratory Medicine, University of Pennsylvania, Philadelphia, PA, USA; ^10^ Center for Neurodegenerative Disease Research, University of Pennsylvania, Philadelphia, PA, USA; ^11^ Department of Artificial Intelligence & Human Health, Nash Family Department of Neuroscience, Ronald M. Loeb Center for Alzheimer's Disease, Friedman Brain Institute, Neuropathology Brain Bank & Research CoRE, Icahn School of Medicine at Mount Sinai, New York, NY, USA; ^12^ Department of Pathology & Laboratory Medicine, University of Pennsylvania Perelman School of Medicine, Philadelphia, PA, USA

## Abstract

**Background:**

Whether primary age‐related tauopathy (PART) is a distinct age‐associated disorder or merely a prodrome of Alzheimer's disease (AD) remains controversial. Both share similar limbic tau but differ in amyloid burden and tau spread. Differential DNA methylation (DNAm) can offers insights into disease biology, biomarkers, and potential therapeutic targets. We therefore used a machine learning classifier trained on DNAm to distinguish PART from AD and then applied the classifier to stratify pathologically‐indeterminate cases.

**Method:**

We evaluated DNAm frontal cortex from ROSMAP (*N* = 707), and trained a support vector machine classifer on 176 PART (A0‐3, B0‐3, C0) and 118 AD (A0‐3, B3, C3) cases. We validated on 142 external cases from the Mount Sinai Brain Bank. We then applied the classifier to stratify neuropathologically‐indeterminate cases (A0‐3, B0‐3, C1‐2 and A0‐3, B0‐2, C3) as Predicted‐PART or Predicted‐AD. We compared the neuropathological, cognitive, DNAm, and transcriptomic profiles of the prediction groups.

**Results:**

When trained on a random sample of 80% of PART and AD cases, the classifier achieved 65% positive and 81% negative predictive value on the remaining 20% of cases. A final model trained on all ROSMAP PART and AD cases accurately classified a majority of external Braak NFT Stage 0‐II cases as Predicted‐PART (63%) and a majority of Braak NFT Stave V‐VI cases as Predicted‐AD (85%). The classifier stratified neuropathologically‐indeterminate cases into prediction groups that had similar tau and amyloid burden, but differed in methylation at 570 CpGs, associated expression of 2,179 genes, and gene ontology terms related to vesicle transport, oxidative phosphorylation, and synaptic transmission. Despite similarities in neuropathological burden, Predicted‐PART individuals scored higher on the MMSE than Predicted‐AD individuals (*p* <1E‐5).

**Conclusions:**

DNAm distinguishes PART from AD. The DNAm‐informed machine learning tool predicts PART vs. AD with high accuracy in multiple cohorts. Moreover, it stratifies indeterminate cases with similar pathology into biologically distinct groups. Together, the data suggest that in individuals with PART, a specific brain epigenetic and biological program contributes to resistance to AD pathology and associated cognitive resilience.